# Reconstruction of Cell Lineage Trees in Mice

**DOI:** 10.1371/journal.pone.0001939

**Published:** 2008-04-09

**Authors:** Adam Wasserstrom, Rivka Adar, Gabi Shefer, Dan Frumkin, Shalev Itzkovitz, Tomer Stern, Irena Shur, Lior Zangi, Shai Kaplan, Alon Harmelin, Yair Reisner, Dafna Benayahu, Eldad Tzahor, Eran Segal, Ehud Shapiro

**Affiliations:** 1 Department of Biological Chemistry, Weizmann Institute of Science, Rehovot, Israel; 2 Department of Computer Science and Applied Mathematics, Weizmann Institute of Science, Rehovot, Israel; 3 Department of Immunology, Weizmann Institute of Science, Rehovot, Israel; 4 Department of Veterinary Resources, Weizmann Institute of Science, Rehovot, Israel; 5 Department of Cell and Developmental Biology, Sackler School of Medicine, Tel-Aviv University, Tel-Aviv, Israel; Baylor College of Medicine, United States of America

## Abstract

The cell lineage tree of a multicellular organism represents its history of cell divisions from the very first cell, the zygote. A new method for high-resolution reconstruction of parts of such cell lineage trees was recently developed based on phylogenetic analysis of somatic mutations accumulated during normal development of an organism. In this study we apply this method in mice to reconstruct the lineage trees of distinct cell types. We address for the first time basic questions in developmental biology of higher organisms, namely what is the correlation between the lineage relation among cells and their (1) function, (2) physical proximity and (3) anatomical proximity. We analyzed B-cells, kidney-, mesenchymal- and hematopoietic-stem cells, as well as satellite cells, which are adult skeletal muscle stem cells isolated from their niche on the muscle fibers (myofibers) from various skeletal muscles. Our results demonstrate that all analyzed cell types are intermingled in the lineage tree, indicating that none of these cell types are single exclusive clones. We also show a significant correlation between the physical proximity of satellite cells within muscles and their lineage. Furthermore, we show that satellite cells obtained from a single myofiber are significantly clustered in the lineage tree, reflecting their common developmental origin. Lineage analysis based on somatic mutations enables performing high resolution reconstruction of lineage trees in mice and humans, which can provide fundamental insights to many aspects of their development and tissue maintenance.

## Introduction

**Figure 1:**
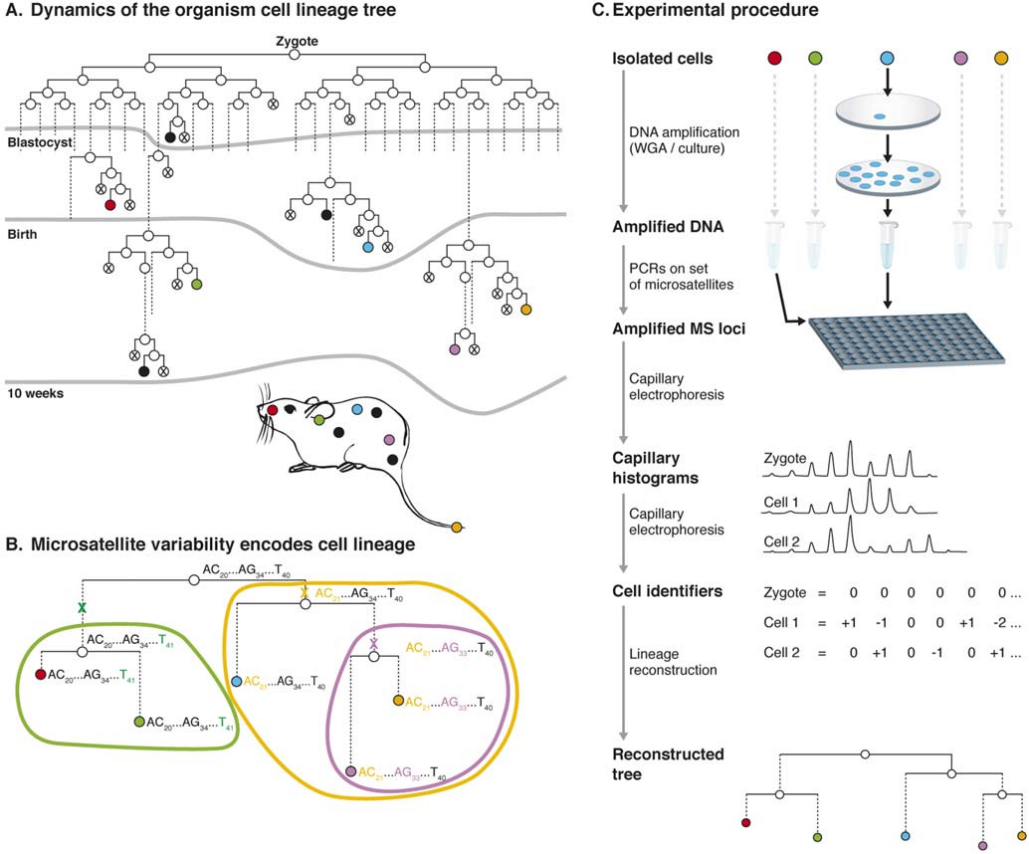
Cell lineage analysis based on somatic MS mutations. A. The cell lineage tree of an organism is a rooted labeled binary tree representing its development from a single cell until present time. Nodes (circles) represent cells (dead cells are crossed), and edges (lines) connect parent and daughter cells (dashed lines represent several divisions). Uncrossed leaves (nodes with no daughters) represent extant cells. Lineage trees are a snapshot of a specific timepoint, which constantly grow throughout embryonic development and the adult life of the organism. Any cell sample (colored leaves) induces a partial subtree, called the cell sample lineage tree (panel B). B. The genomic composition of cells in the sample tree at three MS loci is shown. Spontaneous somatic mutations (marked by X) in these loci are sufficient to encode the lineage relations between these cells. Cells that are genetically similar tend to share a longer common developmental path, consequently enabling phylogenetic analysis to reconstruct the tree. Although idealized mutations are depicted, even potential hampering factors (such as coincident mutations) are not expected to disrupt analysis if a sufficient number of loci are analyzed [1]. C. Scheme of reconstruction procedure. DNA from isolated cells is amplified either by culture or whole genome amplification, after which each cell is analyzed over a large set of MS loci using PCR and capillary electrophoresis. Capillary histograms are automatically analyzed yielding cell identifiers, which represent the mutations each cell accumulated in the MS set. Phylogenetic analysis of cell identifiers yields a reconstructed tree, which is an estimation of the lineage relations between analyzed cells.

All the cells in the body of a multi-cellular organism, such as a human or a mouse, descend from a single cell–the fertilized egg. The exact history of cell divisions that an organism underwent since its beginning is naturally represented by a mathematical tree, which we call the organism cell lineage tree ([1], Figure 1A). Lineage trees encapsulate a wealth of information regarding the development and maintenance of the various subsystems of these organisms under physiological and pathological conditions. Lineage analyses, aiming to elucidate various parts of lineage trees, have been performed until now using a variety of methods. Direct observation of cell divisions enabled the complete reconstruction of the lineage tree of somatic cells of *Caenorhabditis elegans* [2], [3], yet this method is inapplicable to humans and mice since they are opaque and have a tremendous number of cells [4]. A variety of methods for lineage analysis, generally termed clonal assays (reviewed in [5]), rely on marking some cells and tracing their progeny. These methods have yielded many insights, but can provide only course-grain information about a cell lineage tree [1], [4]. In addition, these methods are also inapplicable to the study of humans because they are invasive.

A new approach for cell lineage analysis was recently proposed by our group [1] and later independently by another research group [4], [6]. This approach exploits stochastic processes that occur during the normal development of higher organisms. Before a cell divides, its genome is duplicated with very high precision, yet several mistakes, known as somatic mutations, occur in this process. These mutations are random and sufficiently rare such that they usually do not disrupt the functionality of the cell. Nevertheless, they contain very valuable information, as cells that share a common developmental path tend to share the mutations that occurred along this path. Our analysis shows that in higher organisms such as human and mouse the information available in such somatic mutations is rich enough to implicitly encode the entire cell lineage tree of an organism with very high precision ([1], Figure 1B). The fact that species that share a long evolutionary path tend to have similar genomes compared to species that have diverged earlier in evolution enables phylogenetic analysis at the species level. This similar phenomenon in cells of a multicellular organism enables the application of phylogenetic analysis to reconstruct the lineage relations between cells [1].

Because somatic mutations are relatively rare events, our analysis focuses on microsatellites (MS; repetitive DNA sequences with relatively high mutation rates) in mismatch repair (MMR)-deficient organisms. Such organisms have a much higher rate of MS mutations [7], [8] without compromising their normal development. Another application of this approach was also demonstrated in a 7-month old wild-type mouse in the reconstruction of the lineage relations between about 50 cells (mainly hepatocytes) using fast-mutating MS [6].

Here we applied this method to the study of mouse cell lineage trees. We first aimed to get a glimpse of what mouse lineage trees look like, and to have a sense of their general features, structure and complexity. For this purpose we analyzed multiple cell types obtained from various sources in the mouse body. This analysis also allowed us to address for the first time three basic questions in developmental biology of higher organisms:

1. What is the correlation between lineage and function? (we use the terms cell type and function interchangeably in this paper)
2. What is the correlation between lineage and physical proximity among cells?
3. What is the correlation between lineage and anatomical proximity among cells?

*Caenorhabditis elegans* offers the best point of reference for the first question, since its entire lineage tree (including 959 somatic cells, all with known function) has been reconstructed [2], [3]. Even in *C. elegans*, in which there is a fixed relationship between cell ancestry and cell fate, lineage boundaries do not necessarily coincide with functional boundaries [3]. Two exceptions are its intestine and the germ line, both of which are identifiable tissues generated as “single exclusive clones” [3], i.e. in each case one precursor generates all the cells of the tissue and no other cell. Examples of partial clonal derivation are also present in *C. elegans*: some precursors generate only, but not all, cells of a given type; other precursors generate all, but not only, cells of a given type. However, throughout most of the lineage tree, cell types are intermingled even at the terminal divisions [3]. This is true for various invertebrate taxa, whose lineage trees show little correlation between the phenotypic similarity of two differentiated cells and the closeness of their genealogical relation [9]. While in *C. elegans* and other simple organisms the relation between lineage and function is completely known (though not necessarily understood), this relation is much less known in higher organisms. In order to address this, and other similar questions in mice, we defined a general scheme for lineage analysis which assigns statistical significance to the correlations between lineage and various cell properties (see Results).

In addition to obtaining knowledge regarding the general features of the lineage tree, we also focused on a specific cell type–satellite cells–a small population of muscle stem cells residing beneath the basal lamina of each myofiber [10]. Satellite cells, which are normally quiescent, are activated in response to muscle injury, upon which they proliferate, differentiate and fuse together to repair or replace damaged myofibers [10]. Despite the extensive research, the exact lineage of satellite cells is unknown. We analyzed satellite cells in two respects: first, whether there is a correlation between the lineage of satellite cells and their physical proximity. Specifically, we ask whether cells which are physically closer share a significantly longer common developmental path. In general, sister cells can stay close to each other following cell division, a situation called coherent growth [11] which has been demonstrated in compartments [12] and in cancer [Frumkin D. *et al.*, submitted]. Alternatively, sister cells can disperse as a result of cell motility, a common phenomenon in early embryogenesis of vertebrates [11], for example in the widespread dispersion of neurons across functional regions of the cerebral cortex [13]. Second, we checked whether there is a correlation between the lineage of satellite cells and anatomical proximity. The anatomy of skeletal muscle defines a hierarchy among its components: each muscle is made of many myofibers, on each of which reside several satellite cells. We defined anatomical proximity between satellite cells according to their relation within this hierarchy, and the hierarchy of the body (left vs. right lateralities). We analyzed whether satellite cells from the same myofiber, muscle or body-side have a significantly longer common developmental path than a random pair of cells.

## Results

### Cell Lineage Reconstruction

**Figure 2:**
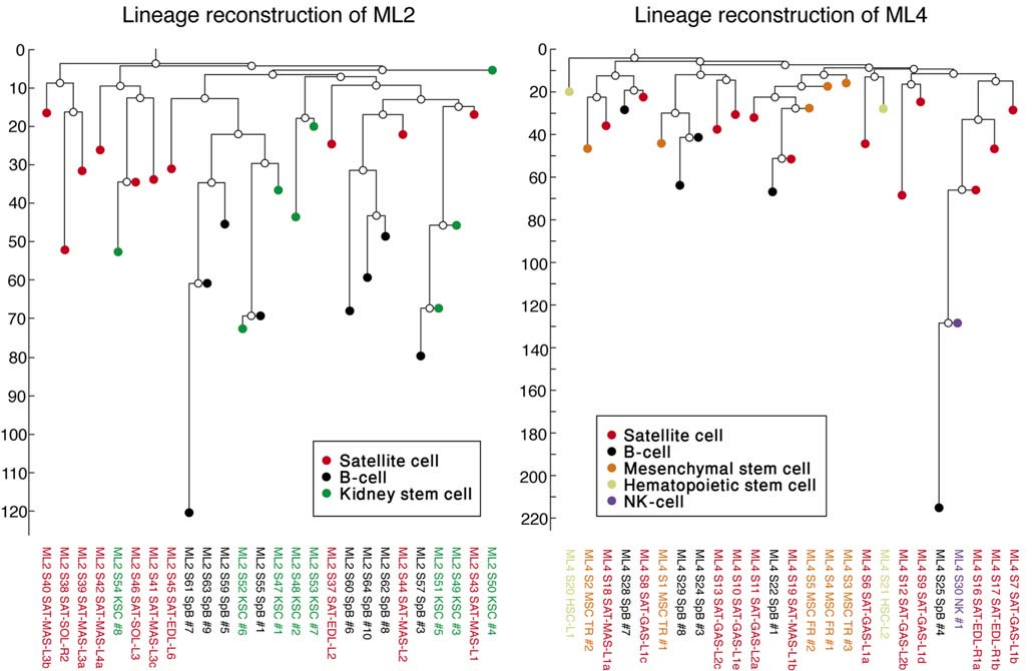
Lineage reconstruction of ML2 and ML4. Reconstructed trees of 26 cells of ML2 (10 week old mouse) and 25 cells of ML4 (13 week old mouse) are shown. Terminal nodes represent single analyzed cells (colors are used for different cell types), while intermediate nodes (uncolored) represent hypothesized precursor cells. Reconstruction was performed using the Neighbor-Joining algorithm (with the distance function ‘Absolute-distance’, see Methods). The vertical axis represents number of cell divisions, and the estimated depth for each cell is obtained by projecting it to this axis. These estimates were obtained by a method we developed, which is based on the correlation between genetic distance and cell depth [15]. Reconstructed trees show that none of the analyzed cell types is completely and solely clustered on a subtree, demonstrating that none of cell types is a single exclusive clones.

**Figure 3:**
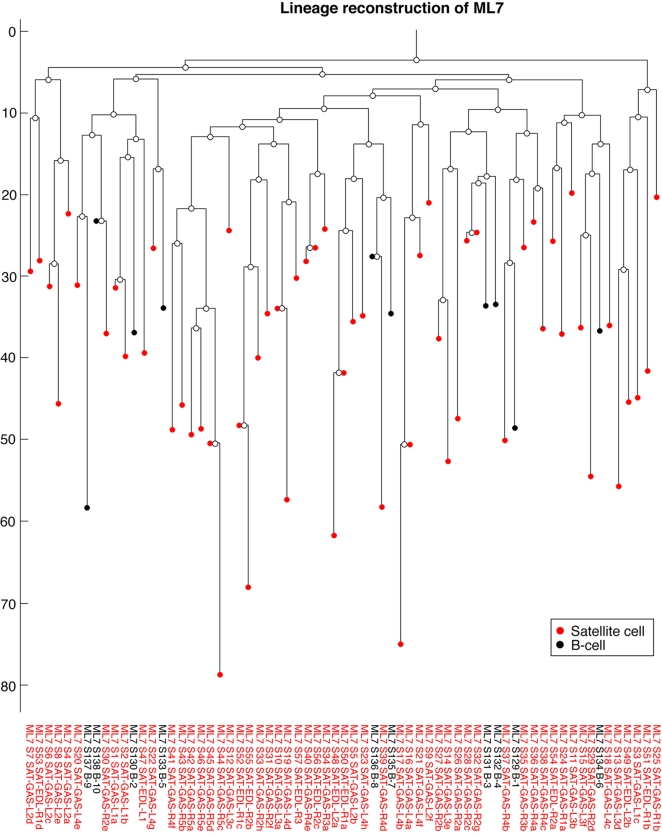
Lineage reconstruction of ML7. Reconstructed tree of 67 cells of ML7 (5.5 week old mouse) is shown (see Figure 2 for explanation of reconstructed trees). This experiment mainly focused on satellite cells (muscle stem cells), which were isolated from various muscles and myofibers from the mouse body. Although no myofiber is a single exclusive clone, we show that satellite cells from the same myofiber have a significantly larger lineage measure, reflecting that they tend to share a longer common developmental path (see Figure 4F).

We performed three lineage experiments on mice called ML2, ML4 and ML7 at the age of 10, 13 and 5.5 weeks respectively. In each experiment, we isolated single cells of multiple types (see complete lists in Tables S1, summarized in Table 1) and performed a semi-automated procedure (similar to that described in [1]) which yields lineage trees depicting their lineage relations (Figure 1C). In these experiments, genomic DNA from each cell was amplified (either by whole genome amplification or *ex vivo* culture) and then genotyped over a set of almost 100 MS loci (of various repeat units and lengths, see Table S2). Genotyping (performed automatically, see Text S1) assigns to each cell a vector, called an identifier [1], representing the mutations it accumulated at the set of loci in comparison to the zygote (see Tables S3a–c for complete cell identifiers; see Figure S1 for examples of MS mutations and signal analysis issues). Tail DNA was used to approximate the putative zygote of each mouse based on the assumption that the tail contains cells of multiple lineages whose most recent common ancestor (MRCA) is most probably the zygote, or one of its immediate descendants. Overall we identified 1156 mutations, accounting for 10.7% of analyzed signals. Cell identifiers served as input to the Neighbor-Joining (NJ) reconstruction algorithm [14] (applying the distance function ‘Absolute-distance’, see Materials and Methods). For analysis we chose informative samples that had at least 5% amplification and at least one mutation (ML7 oocytes were not included in the lineage analysis and will be reported separately with more comprehensive oocyte data). The output of this analysis for each experiment was a rooted binary tree depicting the inferred lineage relations between the set of sampled cells and the zygote, which includes intermediate nodes representing precursor cells of the sample (Figures 2–3). Beyond lineage relations, reconstructed trees also show the estimated depths of individual cells (the depth of a cell is the number of cell divisions it underwent from the zygote). These depths were obtained using a new method we recently developed, based on the correlation between genetic distance and cell depth, using an *ex vivo* cell cultured tree for calibration [15]. While reconstructed trees are enlightening in themselves, some analysis (see below) is required to understand the information they encapsulate.

**Table 1:** Cell Samples in ML Experiments.

Mouse	Cell Type	Source	Expansion	% Mut.	% Amp.
**ML2** (26)	Satellite cell (10)	EDL (2): Left (2 fibers)	Culture	9.8%	85.4%
Age: 10w		SOL (2): Left (1, 1 fiber)			
		Right (1, 1 fiber)			
		MAS (6): Left (4 fibers)			
	Kidney SC (8)	Kidney	Culture	13.4%	38.7%
	B-cell (8)	Spleen	WGA (G-Plex)	18.2%	24.6%
**ML4** (25)	Satellite cell (12)	GAS (8): Left (2 fibers)	Culture	13.3%	69.7%
Age: 13w		EDL (2): Right (1 fiber)			
		MAS (2): Left (1 fiber)			
	Mesenchymal SC (5)	Right Tibia (3)	Culture	8.5%	72.9%
		Right Femur (2)			
	Hematopoietic SC (2)	Left Femur/Tibia	Culture	9.5%	92.8%
	B-cell (5)	Spleen	WGA (G-Plex)	16.3%	24.6%
	NK-cell (1)	Spleen	WGA (G-Plex)	11.1%	13.4%
**ML7** (67)	Satellite cell (57)	GAS (46): Left (23, 4 fibers)	Culture	12.9%	64.0%
Age: 5.5w		Right (23, 5 fibers)			
		EDL (11): Left (3, 2 fibers)			
		Right (8, 3 fibers)			
	B-cell (10)	Spleen	WGA (G-Phi)	13.6%	69.3%

Number of cells in each experiment from each cell type and organ source are indicated in brackets. Only cells which participated in lineage analysis are shown. % Amp. = percent of alleles which were successfully amplified on average from each cell type (based on manual analysis of signals); % Mut. = Percent of mutated alleles with respect to putative zygote (out of amplified alleles). w = weeks; SC = Stem Cell; EDL = Extensor Digitorum Longus muscle; SOL = Soleus muscle; GAS = Gastrocnemius muscle; MAS = Masseter muscle; WGA = Whole Genome Amplification; G-Plex = GenomePlex; G-Phi = GenomiPhi.

### Lineage Analysis of Reconstructed Trees

In this work we perform lineage analysis in an attempt to find correlations between the cell lineage tree and cell type and the anatomical proximity of satellite cells within the hierarchy of muscle. Our analysis tests whether cells within a certain set (e.g. B-cells or satellite cells from the same muscle) have on average a longer common developmental history than a random pair of cells (see Figure S2 for various theoretical outcomes in a lineage analysis checking the correlation between lineage and cell type). We use two measures between a pair of cells that are expected to correlate well with the length of the common developmental history of a pair of cells: the depth of the MRCA of the pair (as assigned by the reconstruction algorithm), and the percent of common mutations shared by the pair. The latter measure does not depend on a reconstructed tree, which has a speculative component in it, but rather on the “raw” data, i.e. the cell identifiers. We performed these analyses only in experiment ML7 because experiments ML2 and ML4 had a small sample of cells.

### Analyzed Cell Types in Mouse are not Single Exclusive Clones

A quick glance at the reconstructed lineage trees of ML2, ML4 and ML7 (Figures 2–3) reveals that none of the analyzed cell types (as given in Table 1) is a single exclusive clone, since no cell type is completely and solely clustered on a subtree. We further investigated whether there may be a subtler correlation between lineage and cell type in ML7. We performed lineage analysis as described above (using both measures) and found that neither of the cell types (satellite cells and B-cells) is significantly correlated to length of common developmental history.

### Satellite Cells within a Myofiber Share a Longer Common Developmental History

**Figure 4:**
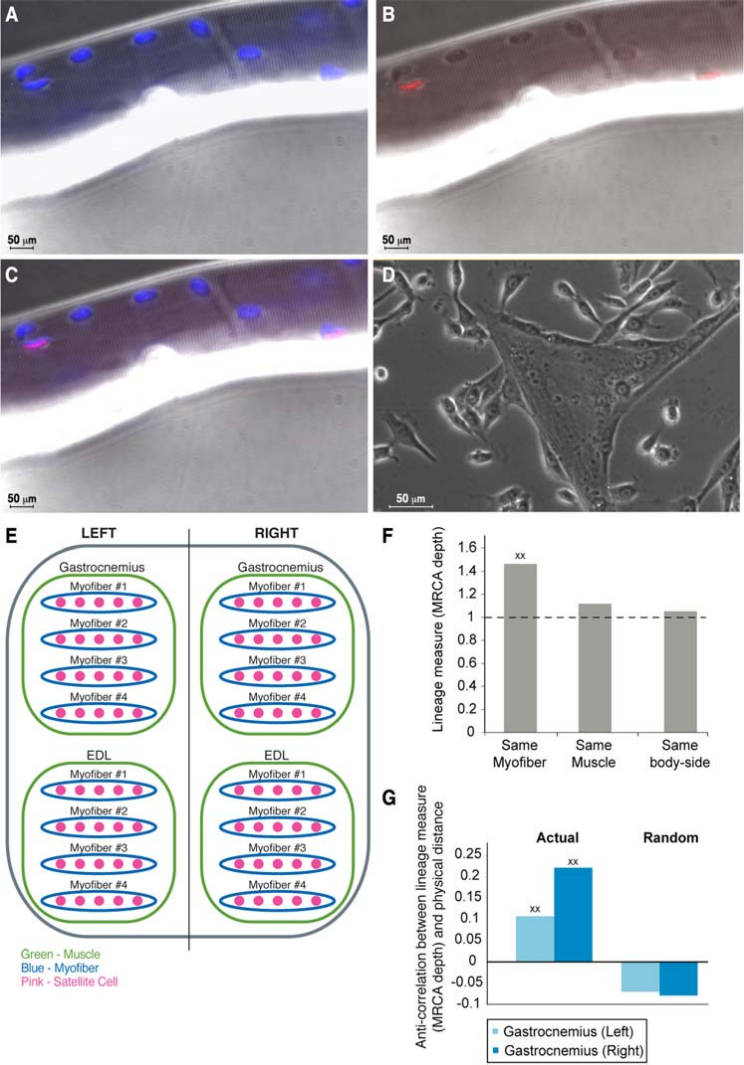
Lineage analysis of satellite cells. A muscle fiber from the Gastrocnemius muscle of ML4 was stained for DAPI (blue, all nuclei within the fiber) and Pax7 (pink, satellite cells). A. DAPI staining. B. Pax7 staining showing two satellite cells. C. Overlay of DAPI and Pax7. D. Single satellite cells were isolated from muscle fibers and cultured *ex vivo*, creating clones whose DNA approximates the DNA of the founder cell. Part of a Petri dish containing a single cell culture is shown. Myofiber structures are evident also in culture. E. Satellite cells can be categorized based on a hierarchy of body-side, muscle and myofiber. Myofibers are numbered from most superficial (#1) until deepest in the muscle. F. The average MRCA depth between pairs of satellite cells from the same myofiber/muscle/body-side was compared to the average MRCA depth between all pairs of cells. Pairs of satellite cells from the same myofiber had on average a significantly larger MRCA depth than a random pair of cells (p<0.0001) demonstrating that myofiber is a significant lineage unit. G. Correlation between lineage and physical proximity in the two main muscles of ML7. The physical distance between myofibers was defined as the difference between myofiber indices (e.g. the distance between a satellite cell on myofiber #3 and a satellite cell on myofiber #1 is 2). Physical distance and MRCA depth are significantly anti-correlated (p = 0.02 and p = 0.0001 for Gastrocnemius left and right, respectively; values stand for anti-correlation coefficients), demonstrating that satellite cells which are physically close to each other tend to share a common developmental path.

The mouse body contains a large number of muscles (on the left or right body side), each of which is made of a bundle of myofibers, in each of which reside about 10–20 satellite cells [16] (Figure 4E). This hierarchy between the various components, which together make skeletal muscle, led us to ask whether satellite cells from the same muscle have a significantly longer common developmental path than satellite cells from different muscles. Satellite cells have yet another advantage which enables performing this type of analysis–they can be isolated from pre-determined physical locations in the mouse while preserving this information for each analyzed cell (Figure 4A–D).

In each experiment we isolated several satellite cells from various muscles and myofibers (see Table 1). We labeled each satellite cell according to its laterality (left or right), muscle identity (e.g. Gastrocnemius), and myofiber (e.g. 2^nd^ myofiber of Gastrocnemius). We analyzed the correlation between length of common developmental history and myofiber, muscle and body-side, as described above (Figure 4F). We found that satellite cells from the same myofiber have a significantly longer common developmental history than a random pair of cells (p<0.0001) using both measures (MRCA depth and percent of common mutations). Satellite cells from the same muscle also have a significantly longer common developmental history than a random pair of cells (p<0.002 for both measures), but this is because of the significance of myofibers (satellite cells from the same myofiber are necessarily also from the same muscle). To test the significance of muscle independent of myofiber, we analyzed satellite cells pairs from the same muscle, but different myofibers. In this case, they did not have a significantly longer common developmental history than a random pair of cells (p>0.32 for both measures). Satellite cell pairs from the same body-side did not show a significantly longer common developmental history than a random pair of cells using any of the measures. Taken together, then results suggest that myofiber is a significant lineage unit in mouse.

### Physically Closer Satellite Cells Share a Longer Common Developmental History

Next, we analyzed whether physically closer satellite cells share a longer common developmental history. Myofibers are numbered according to the order in which they were encountered when penetrating the muscle, from superficial to deepest. This implies that myofibers with adjacent indexes are also close physically. We define the physical distance between two myofibers to be the difference between their indices (e.g. the distance between two satellite cells from the same muscle, one from myofiber #3 and the other from myofiber #1, is 2). We analyzed the two main muscles in ML7: Gastrocnemius-Left (including 23 satellite cells from 4 myofibers) and Gastrocnemius-Right (23 satellite cells from 5 myofibers). The other two muscles (EDL-Left and EDL-Right) were comprised of a small number of satellite cells (3 and 8, respectively) hence we preferred not to analyze them. For each muscle we created a lineage matrix (according to our two measures) and a physical distance matrix (according to the physical distance defined above), and checked whether they are significantly correlated (see Methods). We found that for both muscles there is a significant anti-correlation between the lineage measures and physical proximity (Figure 4G; p = 0.0018 and p = 0.0002 for Gastrocnemius left and right, respectively, according to the MRCA depth measure, and p = 0.0063 and p = 0.0259 according to the percent of common mutations measure). This anti-correlation demonstrates that satellite cells which are physically close to each other tend to share a longer common developmental path, and suggests a relatively coherent growth of satellite cells in myofibers within a muscle.

### Evaluation of DNA Amplification Fidelity

The methods we employed for amplifying DNA from single cells, whether by *ex vivo* culturing or WGA, are not expected to introduce any substantial bias in our analyses, for the following reasons. When single cells are cultured *ex vivo*, mutations may occur during the clonal expansion, yet the identifier of a cell clone is with high probability identical to the identifier of the clone's founder cell [1]. WGA may introduce artifact mutations [17], which cannot be distinguished from mutations that occurred in the mouse. To evaluate the level of WGA artifact mutations we created small colonies, each founded by a single RMA cell [18], and analyzed the cell identifier for each progeny cell (using the same set of MS loci). Progeny cells are assumed to have identical genomes, in the case of which mutations are attributed to WGA artifacts. The two WGA methods we used show 0.9%–1.2% mutations (see Tables S4). Since our MS are highly unstable, mutations may have occurred in culture, hence the actual percent of WGA artifact mutations is expected to be lower. In any case, this figure is significantly lower than the percentage of mutations in the analyzed cells (see Table 1), therefore the majority of mutations occurred *in vivo*.

### Lack of Mutations in ML7 Mesenchymal Stem Cells

In contrast to most cells which showed a high percent of mutations (12.5% on average), 18 out of 20 mesenchymal stem cells (MSCs) in ML7 do not exhibit any mutations over the entire set of analyzed MS (although amplification levels of all cell types amplified in culture were normally high; two MSCs show a few mutations, which may be an artifact due to a slightly different protocol used in their culture, see Materials and Methods). We are currently investigating whether this result is reproducible, and whether it is related to the immortal strand hypothesis [19].

## Discussion

We reconstructed three lineage trees of cells of multiple types obtained from three adult mice based on the analysis of spontaneous somatic mutations accumulated in microsatellites. We analyzed correlations between lineage and cell type, and between lineage and the hierarchy of the anatomy of skeletal muscle. Such correlations are closely related to three basic questions in development: First, are lineage and biological function correlated, i.e. do cells sharing a long common developmental path tend to be of the same type and perform the same biological function? We found that for the cell types we analyzed there is no significant correlation between lineage and cell type. Specifically we showed that none of these cell types are a single exclusive clone. While this is in accordance with the fact that cells in early embryogenesis retain a large developmental potential [20], to the best of our knowledge we are the first to demonstrate it in higher organisms. If any of the cells types were a single exclusive clone, we expect that analysis using our set of MS loci would be sufficient to detect it, since our group was able to detect clonality of tumor cells in distinct physical locations using a similar set of MS [Frumkin D. *et al.,* submitted]. We note three special cases of single exclusive clones that are recognized in vertebrates: cancer, which according to the accepted dogma arises from a single founder cell [21], lymphocytes committed to the production of a particular species of antibody molecule, which arise as a single subclone from a pluripotent lymphocyte [9], and intestinal crypts, which are composed of a monoclonal population of cells descended from a single active multipotent stem cell [22]. The fact that we did not find a correlation between cell type and lineage may be a result of insufficient sampling. Specifically, to detect a significant lineage correlation in a cell population which is polyclonal, one needs to sample at least the number of progenitors of the population (Itzkovitz S. *et al.*, manuscript in preparation). We can provide a lower bound and say that if cell populations such as B-cells sampled in this work arise from several independent clones, the number of clones is higher than the number of cells sampled. In our analysis, we focused on the length of the common developmental history of cells, as measured by MRCA depth in the reconstructed lineage tree and percent of common mutations. Other aspects of cell lineage can be compared as well, such as the path distance between cells in the reconstructed lineage tree. We did not present analysis results that use this measure since we found them to reflect mostly on the depth of cells.

The second and third questions we addressed are related to the development and maintenance of body musculature. We asked whether there is a correlation between cell lineage and physical proximity in satellite cells within a muscle. We found a significant anti-correlation between physical proximity and the length of the common developmental path of these cells. This demonstrates that satellite cells from the same muscle which are physically closer to each other tend to share a longer common developmental path. Next, we asked whether there is a correlation between cell lineage and anatomical proximity among cells, based on the hierarchy of satellite cell-myofiber-muscle-body side. We found that satellite cells from the same myofiber are significantly clustered together on the lineage tree, suggesting they are created (and perhaps maintained) from a small pool of progenitor cells. Our data suggests that satellite cells from the same myofiber are not single exclusive clones because no myofiber is clustered on a distinct subtree. This means that satellite cells from a single myofiber are either created by more than one founder cell during embryogenesis, or become heterogenic during the process of muscle repair following injury. These results may help understand the origins of satellite cells. Our data are most consistent with satellite cells being created locally and temporally during embryogenesis [23]–[25], rather than having a heterogenic origin from various tissues such as the bone marrow or other sources [26], [27]. Our results are currently inconclusive regarding the correlation between muscle and cell lineage. On one hand, we found a significant correlation between physical proximity and depth of common ancestor within a muscle, suggesting that myofibers within muscles are also linked lineage-wise. On the other hand, satellite cells from the same muscle did not have longer common developmental history compared to satellite cells from different muscles. While these findings do not necessarily contradict, a conclusive statement in this respect requires further experiments with more muscles at various physical distances from each other (in ML7, which was our major experiment in satellite cells, we analyzed only two muscles, which were quite close physically). Regarding the correlation between cell lineage and body-side, our results demonstrate that there is no significant correlation between these, at least in satellite cells. In principle, it may be possible to reconstruct the entire satellite cell lineage, which would give a clearer picture regarding the development and maintenance of satellite cells, and should reveal their heterogenic nature.

We have evidence that our method for reconstructing cell lineages from somatic mutations is informative and discovers relations between cells when these exist. First, we found that satellite cells from the same myofiber tend to share a longer common developmental path, which makes sense biologically. Second, our group analyzed cancerous and non-cancerous cells from an older mouse using our method and found that the tumor initiated from a single founder cell approximately five months prior to diagnosis and that the tumor grew in a physically coherent manner (Frumkin *et al.*, submitted). These findings are in accordance with the accepted paradigm of cancer development. Third, our previous work [1] demonstrated an *ex vivo* proof of concept for the method. Extrapolation from *in vitro* to *in vivo* assumes that somatic mutations in MS occur similarly *ex vivo* and *in vivo*.

Beyond the specific biological findings, we see the importance of this work as a demonstration of the potential of this new methodology of lineage analysis. Previous lineage analyses based on classical techniques (such as marking a cell and following its progeny) were unable to reconstruct lineage trees at the single cell level due to inherent limitations. This is now changed: in principle the lineage relations between any set of isolated cells can be reconstructed. The lineage trees shown here as well as the one reconstructed by Horwitz and colleagues [6] are just first examples, but we believe that a new field of cell lineage analysis will emerge. This possibility is also due to technological advancements: WGA of single cells provides large DNA quantities from practically any desired cell [28], and high-throughput DNA analysis technologies enable cheaper and quicker analysis of multiple loci obtained from WGA. In the long term we believe that this methodology and technology will drive large-scale projects such as the “Human Cell Lineage Project” (and its Mouse parallel [1]), aiming to understand ever larger portions of these organisms. We also foresee that this methodology will drive a conceptual change and create a new field of computational lineage analysis. The conceptual change is first of all based on the definition of a cell lineage tree (see [1] and this paper), which defines the lineage relations between cells according to their genealogical relations in this tree. The term lineage has acquired over time multiple meanings [29], which do not necessarily correspond to the rigorous definition of genealogical lineage. For example, it is used to define the acquisition of a state of commitment (e.g. ‘B-lymphocyte lineage [30]) or a specific tissue of origin (e.g. ‘mesodermal lineage’ [31]). Among developmental biologists, such distinctions are not always made explicit or even recognized [32]. Computational analyses of cell lineage trees coupled to biological or clinical understanding of these analyses are required to understand such trees. These analyses are necessary because lineage trees encapsulate a wealth of information, yet most of this information is not self-evident. Understanding the cell lineage trees of higher organisms, especially human, is a fundamental challenge of many branches of biology and medicine (see [1]). Several open questions are actually lineage questions in disguise, and knowledge of the relevant portions of the cell lineage tree could shed light on these questions. For example, in cancer research, lineage analysis can reveal the timing of tumor initiation, the relation between metastases and primary tumors, and the differential response of tumor subpopulation to cancer therapy. In stem cell research, depth analysis at successive timepoints can reveal the number of stem cells maintaining a niche, and their dynamics in physiological and pathological states. In developmental biology research, lineage analysis can reveal the developmental origin of various tissues. Here we performed a few such analyses, but we believe this is only the tip of the iceberg, and further analysis may yield deeper insights. For example, by applying tools and concepts used in the analysis of human populations to cell populations, it may be possible to derive quantitative estimates regarding the cell dynamics and the number of stem cells which maintain a tissue. Perhaps most importantly, the fact that this methodology is non-invasive theoretically allows analyzing humans, without being confined to analysis of model organisms.

## Materials and Methods

### Experiment mice

Mlh1+/− mice were obtained from Michael Liskay (described in [33]) and were maintained at our institute under C57Bl/6 and 129SvEv (kindly provided by Ari Elson, The Weizmann Institute of Science) backgrounds. Mlh1+/−C57Bl/6 and Mlh1+/− 129SvEv were mated to yield Mlh1−/− progeny of dual background, which were used for experiments. ML2, ML4 and ML7 were 10, 13 and 5.5 weeks old respectively when sacrificed, and were genotyped as Mlh1−/−. All animal husbandry and euthanasia procedures were performed in accordance with the Institutional Animal Care and Use Committee at the Weizmann Institute of Science.

### Isolation and culture of satellite cells

Satellite cells were isolated and cultured as described in [34]. Briefly, EDL, soleus, gastrocnemius and masseter muscles were digested in 0.2% (w/v) collagenase type I (Sigma-Aldrich) at 37°C. Collagenase was reconstituted in Dulbecco's Modified Essential Medium (DMEM; high glucose, with L-glutamine, 110 mg/l sodium pyruvate, and pyridoxine hydrochloride; supplemented with 50 U/ml penicillin and 50 mg/ml streptomycin; GIBCO Invitrogen). Following digestion, the muscles were triturated with a wide-bore pipette to release single myofibers. Each single myofiber was transferred to a separate 60 ml dish and then to a tube containing 1ml DMEM. Single myofibers were triturated using a 20G needle mounted onto a 1 ml syringe, to disengage satellite cells. The resulting fiber suspension (in 1 ml DMEM) was then dispensed to 12 Matrigel pre-coated wells within a 24-well plate. Clones were observed every other day.

### Isolation and culture of mesenchymal stem cells (MSCs)

MSCs were isolated and cultured as described in [35], [36]. Briefly, femurs were cleaned off the soft tissue and epiphysis to allow bone marrow cells (BMC) collection. The BMC were flushed out with DMEM using syringe with 21G needle. To get Colony forming unit-fibroblast (CFU-F) single cell BMC suspensions were diluted to a concentration of 2.5×10^6^ cells/ml in DMEM supplemented with 10% FCS and plated in 24-wells plates. MSC clones from ML7 samples 104 and 105 were created slightly differently: due to uncertainty of a single cell origin of these clones, a second subcloning step was performed, and cells were allowed to proliferate for additional 4–5 days.

### Isolation and culture of kidney stem cells

Kidney stem cells were isolated and cultured as described in [37].

### Isolation and culture of hematopoietic stem cells

Hematopoietic stem cells (SCA-1^+^ Lin^−^) were obtained by crushing tibiae and femurs and extracting cells from the bone marrow (BM). The BM cells suspension were passed through a nylon mesh twice to remove connective tissue and clumps of cells. Subsequently, the cells (20×10^6^) were washed and resuspended at 5ml PBS. BM cell suspensions were maintained on ice throughout the purification procedures. Antibodies used for immunomagnetic positive selection were SCA-1 conjugated to microbeads (SCA-1 multisort kit, Miltenyi Biotec, Bergisch Gladbach, Germany). The following lineage marker antibodies conjugated to microbeads were used: CD45R/B220 for the B lineage, CD4/L3T4 and CD8a/Ly-2 for T cell lineage, and CD11b/Mac-1 for myelomonocytic cells. SCA-1^+^ cells were positively selected using the MACS® magnetic bead system (Miltenyi). The microbeads were removed from the magnetic field, thereby allowing the SCA-1^+^ cells to be collected into the multisort release reagent (Miltenyi). Cytofluorimetric sorting of the isolated SCA-1^+^ cells was carried out by double immunofluorescent staining, using the following directly labeled antibodies (obtained from Pharmingen, San Diego, CA): PE-SCA-1/Ly-6A/E (clone E13-161.7), R-APC-CD45R/B220 (clone RA3-6B2), R-APC-CD4/L3T4 (clone GK 1.5), R-APC-CD8a/Ly-2 (clone 53-6.7) and R-APC-CD11b (clone M1/70). Stained cells were resuspended at 1×10^6^ cells /ml in buffer column at 4°C (on ice). Cells were then sorted for positive SCA-1-PE cells and lineage negative cells-APC (SCA-1^+^ Lin^−^ cells) into a sterile glass tube containing 500 µl/ml FCS sterile using ARIA sorter (BD Biosciences, Mountain View, CA). Immediately after purification, 10^3^ cells per ml SCA-1^+^Lin^−^ cells were suspended in 10 ml of culture medium. Cells were counted and diluted to reach an average of 1 cell/600 µl culture medium and replated into 96-well culture plates (200 µl/well) to establish single cell clones. Single cell clones were cultured for 7 days in 96-wells plate (Nunc, Roskilde, Denmark) in culture medium made with Iscoves Modified Dulbeccos Medium (IMDM) containing 10% FCS, 2 mM L-glutamine, 100 U/ml penicillin and 0.1 mg/ml streptomycin.

### Isolation of single cells from a suspension

Single cells were isolated by suspending a bulk of cells in PBS until a dilution of about 1 cell/0.5 µl was obtained. Then, 0.5 µl drops of this dilution were placed at the center of multiple wells of a 96-well (flat bottom) plate. Microscopic observation was used to identify wells with exactly one cell.

### Isolation of B-Cells, NK-Cells

Spleens of experiment mice were dissected and crushed over a 1 µm mesh (A.D. Sinun, Israel) into a Petri dish obtaining a cell suspension. The cell suspension was transferred into a 50 ml tube, centrifuged (7 min, 1200 rpm) in a 5702 Eppendorf centrifuge. Cells were resuspended in 1 ml PBS and counted. Isolation of specifically desired cells was performed by magnetic sorting using the Miltenyi Biotec magnetic microbeads, MACS columns and MACS separator, according to the manufacturers instructions. CD45R (B220) microbeads were used for B cells, and CD49b (DX5) microbeads were used for NK cells. Single B-cells and NK-cells were obtained as described above.

### Isolation of oocytes

Ovaries were removed and placed in Leibovitz's L-15 tissue culture medium (Gibco), supplemented with 5% fetal bovine serum (Biolab, Jerusalem, Israel), penicillin (100 IU/ml) and streptomycin (100 µg/ml, Gibco). When isolated from the ovarian follicles, the oocytes were arrested at the first prophase. To maintain meiotic arrest in fully grown oocytes, the phosphodiesterase inhibitor, isobutylmethylxantine (IBMX) (0.2 mM, Sigma), that prevents cAMP degradation was included in the medium of incubation [38]. The follicles were punctured under a stereoscopic microscope in order to release the cumulus–oocyte complexes that were then placed into acidic L-15 medium (pH 6.0) to obtain cumulus-free oocytes. Each oocyte was placed in a 0.2 mL tube (ABgene) in a volume of 5 µL medium. Oocytes were frozen in liquid nitrogen and kept in −80°C until analyzed.

### Whole Genome Amplification of single cells

was performed using the GenomePlex Single Cell Whole Genome Amplification Kit (Sigma #WGA4) and the GenomiPhi DNA amplification kit (GE Healthcare, UK). The GenomePlex kit was used according to the manufacturer's instructions. Prior to the GenomiPhi protocol, cell denaturation was performed as follows: one part (2 µl) of lysis solution (400 mM KOH, 100 mM DTT and 10 mM EDTA) was added to 0.5 ml tubes, each containing a single cell in 2 µl double distilled water. Cells were lysed for 10 min on ice followed by addition of one part (2 µl) of neutralization solution (400 mM HCl, 600 mM Tris HCl, pH 6–prepared by mixing 4 ml of 1 M HCl and 6 ml of 1 M Tris HCl, pH 7.5). At this point each cell was in 6 µl solution (instead of 1 µl according to the protocol). The GenomiPhi protocol was carried out according to the manufacturer's instructions, except that amounts of all the ingredients were multiplied by six.

### Cell identifiers

Because analysis of multiple loci from single cells requires a minimal amount of DNA (typically ∼1 µg in our case) we first amplified genomic DNA either by ex vivo culturing of single cells or by WGA (as described above), or a combination of these. DNA was extracted from cell clones using the Wizard SV Genomic DNA Purification System (Promega). All Primers were obtained from Applied Biosystems. Some were part of the ABI PRISM® Mouse Mapping Primers v.1.0, and others were designed by us using Primer3 (http://frodo.wi.mit.edu/). In each amplification reaction 4 MS loci were amplified together in 25 µl including DNA (25–50 ng when extracted from cell clones, or about 1% of WGA products), 0.2 µM of each primer, 0.2 mM of each dNTP (BIOLINE), and 0.625U of Thermo-Start DNA Polymerase (ABgene). Thermal cycling conditions were: (i) 15’ 95°c, (ii) 35 cycles: 1’ 95°c, 1’ 58°c, 1’ 72°c, (iii) 15’ 72°c. Amplified products from three PCR reactions were combined and run on an ABI prism 3130xl Genetic Analyzer machine (Applied Biosystems). Fragment analysis was performed using the GeneMapper v3.7 software accompanying the machine. We used a programmable laboratory robot (TECAN Genesis) augmented with a PCR machine (Biometra TRobot) to perform the liquid handling for PCR, the PCR itself, and the sample preparation for the capillary machine. Capillary signals were analyzed using an automatic signal analysis program that we designed (see Text S1). Manual override was used in about 1.5% of the signals that were defined by the algorithm as problematic, as well as all signals of samples which were amplified using Genome-Plex, due to their high stutter patterns. Analysis assigns to each MS allele in every sample a relative allelic value–a whole number equal to the difference between the number of repeats of that allele and the number of repeat units of the corresponding allele in the zygote (MS slippage mutations tend to insert or delete repeated units). All relative allelic values together compose the identifier ascribed to each cell.

### Evaluation of whole genome amplification fidelity

RMA cells (kindly donated by Lea Eisenbach, The Weizmann Institute of Science) were maintained in RPM1640 medium (GIBCO, Invitrogen, UK) supplemented with 10% heat inactivated (65°C, 30 minutes) FBS (GIBCO) and 1% Penicillin/Streptomycin (GIBCO). Single RMA cells were obtained as described above, and were allowed to grow for a few days, until clones with about 10–20 cells were obtained. These clones were then separated to single cells, whose identifiers were obtained as described above. Mutations in two loci were overridden as problematic signals (see shaded ‘PS’ signals in Tables S4a–b, discussed in Text S1). Separate experiments were performed for each WGA kit (GenomiPhi and GenomePlex). In the GenomiPhi experiment two clones were created and five progeny cell were analyzed from each clone. This experiment showed 0.9% mutations. In the GenomePlex experiment three clones were created and eight progeny cells were analyzed from one clone, and one progeny cell was analyzed from each of the other two clones. This experiment showed 1.2% mutations. Bulk of RMA DNA was used as a reference against which mutations were assigned.

### Lineage reconstruction

Lineage tree reconstruction was performed with the NJ algorithm (implemented in MATLAB). We used the distance function “Absolute-distance”, in which the distance between two samples is the average distance between their allelic values in all alleles which were analyzed in both samples. The un-rooted output tree was rooted from the node corresponding to the root, yielding the reconstructed rooted tree.

### Lineage analysis of reconstructed trees

Our general scheme for lineage analysis is as follows: (i) a set of cells for which we would like to check their correlation to lineage is chosen, (ii) a lineage measure is chosen, (iii) the ratio between the average lineage measure for all pairs of cells in the set and the average lineage measure for all pairs of cells is calculated. If this ratio is larger (or smaller, depending on the measure) than 95% of the ratios in 10,000 randomizations of the samples, we define the correlation between lineage and this set to be significant. In the analysis of myofibers and muscles we used the same scheme, but compared all pairs of cells within a myofiber/muscle to all pairs of cells in the experiment. We calculated the average lineage measure within all pairs of satellite cells which are on the same myofiber (or muscle), and proceeded as above.

### Analysis of lineage vs. physical proximity in satellite cells

For each analyzed muscle we calculated a lineage matrix and a physical matrix between all satellite cells within the muscle. Each entry in the lineage matrix is the lineage measure between the pair of corresponding cells. Each entry in the physical matrix is the physical distance between the pair of corresponding cells, as defined in the paper (difference between myofiber indices). An anti-correlation coefficient (equal to minus the correlation coefficient) was obtained for each lineage and physical matrix, and was compared to 10,000 randomizations of the lineage matrix. Lineage and physical proximity within a muscle were defined as significantly anti-correlated if the anti-correlation coefficient for the actual data was larger than 95% of the anti-correlation coefficients in the randomizations.

## Supporting Information

Figure S1 Examples of MS mutations and signal analysis issues
(0.04 MB DOC)

Figure S2 Correlation between lineage and cell type
(0.04 MB DOC)

Table S1 List of ML2, ML4 and ML7 cells
(0.26 MB DOC)

Table S2 List of MS Loci used for ML experiments
(0.17 MB DOC)

Table S3 Cell identifiers for ML2
(0.10 MB XLS)

Table S4 Cell identifiers for ML4
(0.10 MB XLS)

Table S5 Cell identifiers for ML7
(0.31 MB XLS)

Table S6 Cell identifiers for WGA fidelity experiments (GenomiPhi)
(0.06 MB XLS)

Table S7 Cell identifiers for WGA fidelity experiments (GenomePlex)
(0.09 MB XLS)

Text S1 Algorithm for automatic analysis of capillary electrophoresis signals
(0.32 MB DOC)
