# Supplementary material for: Reconstruction of Cell Lineage Trees in Mice
Source: PLoS One. 2008 Apr 9;3(4):e1939. doi: 10.1371/journal.pone.0001939 (PMC2276688; doi:10.1371/journal.pone.0001939)

**Figure S1. Examples of MS mutations and signal analysis issues.** ML mice were created by mating pure-bred C57Bl/6 and 129SvEv mice, hence each locus has an allele from each mouse line. Due to mutations in MS most loci are heterozygous, almost doubling the amount of information from such loci. Allelic values of the zygote are zero by definition and relative allelic values of sample alleles are whole numbers equal to the difference between the number of repeats of the sample and the zygote. Analysis of loci with far alleles (ABI73) is relatively simple. Signal analysis assumes minimal number of mutations between possible assignments (e.g. [+1,-1] in ML7 S1 is preferred over [+2,-2]). Analysis of mutations in homozygous loci (D1Mit1001) is problematic since we do not know which allele mutated ([-1,0] and [0,-1] are both plausible). Such loci can disrupt lineage analysis, but are still useful for depth analysis. Analysis of heterozygous loci with a single repeat unit difference between alleles (ABI30) is ambiguous in samples displaying a single allele, as both a mutation and allele dropout (denoted as ‘X’) are possible. In general, we preferred to assign mutations only when we were certain about them.


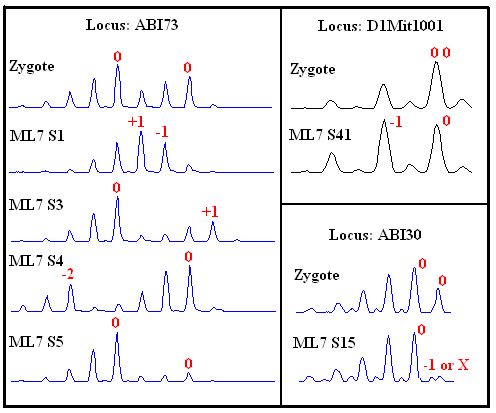

Supplement: Figure S1 — Examples of MS mutations and signal analysis issues (0.04 MB DOC) [file pone.0001939.s001.doc]
