# Supplementary material for: Reconstruction of Cell Lineage Trees in Mice
Source: PLoS One. 2008 Apr 9;3(4):e1939. doi: 10.1371/journal.pone.0001939 (PMC2276688; doi:10.1371/journal.pone.0001939)
Supplement: Figure S2 — Correlation between lineage and cell type (0.04 MB DOC) [file pone.0001939.s002.doc]

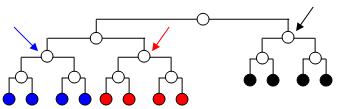


**Figure S2. Correlation between lineage and cell type.** Various theoretical possibilities for the correlation between lineage and cell type are shown. Each cell type is represented by a different color. Case 1 is an extreme case in which each cell type is a single exclusive clone, i.e. for each cell type one precursor (marked by arrows) generates all the cells of the tissue and no other cell. In case 2 no cell type is a single exclusive clone, but there is still a significant correlation between lineage and cell type. In case 3 there is no significant correlation between lineage and cell type.

**(1) Single exclusive clones**


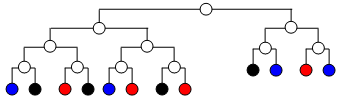

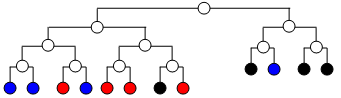


**(2) Significant correlation**

**(3) Heterogeneous**
