# Supplementary material for: Reconstruction of Cell Lineage Trees in Mice
Source: PLoS One. 2008 Apr 9;3(4):e1939. doi: 10.1371/journal.pone.0001939 (PMC2276688; doi:10.1371/journal.pone.0001939)
Supplement: Table S1 — List of ML2, ML4 and ML7 cells (0.26 MB DOC) [file pone.0001939.s003.doc]

**Table S1a. List of ML2 cells**

| **ML2 (10 weeks, 28 samples)** | | | |
| --- | --- | --- | --- |
| **Name** | **Description** | **Source** | **Used in lineage analysis** |
| ML2_S37 | Satellite Cell: EDL-L 2 | EDL muscle (left) | Yes |
| ML2_S38 | Satellite Cell: SOL-R 2 | Soleus muscle (right) | Yes |
| ML2_S39 | Satellite Cell: MAS-L 3a | Masseter muscle (left) | Yes |
| ML2_S40 | Satellite Cell: MAS-L 3b | Masseter muscle (left) | Yes |
| ML2_S41 | Satellite Cell: MAS-L 3c | Masseter muscle (left) | Yes |
| ML2_S42 | Satellite Cell: MAS-L 4a | Masseter muscle (left) | Yes |
| ML2_S43 | Satellite Cell: MAS-L 1 | Masseter muscle (left) | Yes |
| ML2_S44 | Satellite Cell: MAS-L 2 | Masseter muscle (left) | Yes |
| ML2_S45 | Satellite Cell: EDL-L 6 | EDL muscle (left) | Yes |
| ML2_S46 | Satellite Cell: SOL-L 3 | Soleus muscle (left) | Yes |
| ML2_S47 | Kidney stem cell #1 | Kidney | Yes |
| ML2_S48 | Kidney stem cell #2 | Kidney | Yes |
| ML2_S49 | Kidney stem cell #3 | Kidney | Yes |
| ML2_S50 | Kidney stem cell #4 | Kidney | Yes |
| ML2_S51 | Kidney stem cell #5 | Kidney | Yes |
| ML2_S52 | Kidney stem cell #6 | Kidney | Yes |
| ML2_S53 | Kidney stem cell #7 | Kidney | Yes |
| ML2_S54 | Kidney stem cell #8 | Kidney | Yes |
| ML2_S55 | Splenic B-cell #1 | Spleen | Yes |
| ML2_S56 | Splenic B-cell #2 | Spleen | No |
| ML2_S57 | Splenic B-cell #3 | Spleen | Yes |
| ML2_S58 | Splenic B-cell #4 | Spleen | No |
| ML2_S59 | Splenic B-cell #5 | Spleen | Yes |
| ML2_S60 | Splenic B-cell #6 | Spleen | Yes |
| ML2_S61 | Splenic B-cell #7 | Spleen | Yes |
| ML2_S62 | Splenic B-cell #8 | Spleen | Yes |
| ML2_S63 | Splenic B-cell #9 | Spleen | Yes |
| ML2_S64 | Splenic B-cell #10 | Spleen | Yes |

**Table S1b. List of ML4 cells**

| **ML4 (13 weeks, 31 samples)** | | | |
| --- | --- | --- | --- |
| **Name** | **Description** | **Source** | **Used in lineage analysis** |
| ML4_S1 | Mesenchymal SC TR1 | Tibia (right) | Yes |
| ML4_S2 | Mesenchymal SC TR2 | Tibia (right) | Yes |
| ML4_S3 | Mesenchymal SC TR3 | Tibia (right) | Yes |
| ML4_S4 | Mesenchymal SC FR1 | Femur (right) | Yes |
| ML4_S5 | Mesenchymal SC FR2 | Femur (right) | Yes |
| ML4_S6 | Satellite cell: GAS-L1a | Gastrocnemius muscle (left) | Yes |
| ML4_S7 | Satellite cell: GAS-L1b | Gastrocnemius muscle (left) | Yes |
| ML4_S8 | Satellite cell: GAS-L1c | Gastrocnemius muscle (left) | Yes |
| ML4_S9 | Satellite cell: GAS-L1d | Gastrocnemius muscle (left) | Yes |
| ML4_S10 | Satellite cell: GAS-L1e | Gastrocnemius muscle (left) | Yes |
| ML4_S11 | Satellite cell: GAS-L2a | Gastrocnemius muscle (left) | Yes |
| ML4_S12 | Satellite cell: GAS-L2b | Gastrocnemius muscle (left) | Yes |
| ML4_S13 | Satellite cell: GAS-L2c | Gastrocnemius muscle (left) | Yes |
| ML4_S14 | Satellite cell: GAS-L3a | Gastrocnemius muscle (left) | No |
| ML4_S15 | Satellite cell: GAS-L3b | Gastrocnemius muscle (left) | No |
| ML4_S16 | Satellite cell: EDL-R1a | EDL muscle (right) | Yes |
| ML4_S17 | Satellite cell: EDL-R1b | EDL muscle (right) | Yes |
| ML4_S18 | Satellite cell: MAS-L1a | Masseter muscle (left) | Yes |
| ML4_S19 | Satellite cell: MAS-L1b | Masseter muscle (left) | Yes |
| ML4_S20 | HSC #1 | Femur/Tibia (left) | Yes |
| ML4_S21 | HSC #2 | Femur/Tibia (left) | Yes |
| ML4_S22 | B-cell #1 | Spleen | Yes |
| ML4_S23 | B-cell #2 | Spleen | No |
| ML4_S24 | B-cell #3 | Spleen | Yes |
| ML4_S25 | B-cell #4 | Spleen | Yes |
| ML4_S26 | B-cell #5 | Spleen | No |
| ML4_S27 | B-cell #6 | Spleen | No |
| ML4_S28 | B-cell #7 | Spleen | Yes |
| ML4_S29 | B-cell #8 | Spleen | Yes |
| ML4_S30 | NK-cell #1 | Spleen | Yes |
| ML4_S31 | NK-cell #2 | Spleen | No |

**Table S1c. List of ML7cells**

| **ML7 (5.5 weeks, 138 samples)** | | | |
| --- | --- | --- | --- |
| **Name** | **Description** | **Source** | **Used in lineage analysis** |
| ML7_S1 | Satellite cell: GAS-L1a | Gastrocnemius muscle (left) | Yes |
| ML7_S2 | Satellite cell: GAS-L1b | Gastrocnemius muscle (left) | Yes |
| ML7_S3 | Satellite cell: GAS-L1c | Gastrocnemius muscle (left) | Yes |
| ML7_S4 | Satellite cell: GAS-L2a | Gastrocnemius muscle (left) | Yes |
| ML7_S5 | Satellite cell: GAS-L2b | Gastrocnemius muscle (left) | Yes |
| ML7_S6 | Satellite cell: GAS-L2c | Gastrocnemius muscle (left) | Yes |
| ML7_S7 | Satellite cell: GAS-L2d | Gastrocnemius muscle (left) | Yes |
| ML7_S8 | Satellite cell: GAS-L2e | Gastrocnemius muscle (left) | Yes |
| ML7_S9 | Satellite cell: GAS-L2f | Gastrocnemius muscle (left) | Yes |
| ML7_S10 | Satellite cell: GAS-L3a | Gastrocnemius muscle (left) | Yes |
| ML7_S11 | Satellite cell: GAS-L3b | Gastrocnemius muscle (left) | Yes |
| ML7_S12 | Satellite cell: GAS-L3c | Gastrocnemius muscle (left) | Yes |
| ML7_S13 | Satellite cell: GAS-L3d | Gastrocnemius muscle (left) | Yes |
| ML7_S14 | Satellite cell: GAS-L3e | Gastrocnemius muscle (left) | Yes |
| ML7_S15 | Satellite cell: GAS-L3f | Gastrocnemius muscle (left) | Yes |
| ML7_S16 | Satellite cell: GAS-L4a | Gastrocnemius muscle (left) | Yes |
| ML7_S17 | Satellite cell: GAS-L4b | Gastrocnemius muscle (left) | Yes |
| ML7_S18 | Satellite cell: GAS-L4c | Gastrocnemius muscle (left) | Yes |
| ML7_S19 | Satellite cell: GAS-L4d | Gastrocnemius muscle (left) | Yes |
| ML7_S20 | Satellite cell: GAS-L4e | Gastrocnemius muscle (left) | Yes |
| ML7_S21 | Satellite cell: GAS-L4f | Gastrocnemius muscle (left) | Yes |
| ML7_S22 | Satellite cell: GAS-L4g | Gastrocnemius muscle (left) | Yes |
| ML7_S23 | Satellite cell: GAS-L4h | Gastrocnemius muscle (left) | Yes |
| ML7_S24 | Satellite cell: GAS-R1a | Gastrocnemius muscle (right) | Yes |
| ML7_S25 | Satellite cell: GAS-R1b | Gastrocnemius muscle (right) | Yes |
| ML7_S26 | Satellite cell: GAS-R2a | Gastrocnemius muscle (right) | Yes |
| ML7_S27 | Satellite cell: GAS-R2b | Gastrocnemius muscle (right) | Yes |
| ML7_S28 | Satellite cell: GAS-R2c | Gastrocnemius muscle (right) | Yes |
| ML7_S29 | Satellite cell: GAS-R2d | Gastrocnemius muscle (right) | Yes |
| ML7_S30 | Satellite cell: GAS-R2e | Gastrocnemius muscle (right) | Yes |
| ML7_S31 | Satellite cell: GAS-R2f | Gastrocnemius muscle (right) | Yes |
| ML7_S32 | Satellite cell: GAS-R2g | Gastrocnemius muscle (right) | Yes |
| ML7_S33 | Satellite cell: GAS-R2h | Gastrocnemius muscle (right) | Yes |
| ML7_S34 | Satellite cell: GAS-R3a | Gastrocnemius muscle (right) | Yes |
| ML7_S35 | Satellite cell: GAS-R3b | Gastrocnemius muscle (right) | Yes |
| ML7_S36 | Satellite cell: GAS-R4a | Gastrocnemius muscle (right) | Yes |
| ML7_S37 | Satellite cell: GAS-R4b | Gastrocnemius muscle (right) | Yes |
| ML7_S38 | Satellite cell: GAS-R4c | Gastrocnemius muscle (right) | Yes |
| ML7_S39 | Satellite cell: GAS-R4d | Gastrocnemius muscle (right) | Yes |
| ML7_S40 | Satellite cell: GAS-R4e | Gastrocnemius muscle (right) | Yes |
| ML7_S41 | Satellite cell: GAS-R4f | Gastrocnemius muscle (right) | Yes |
| ML7_S42 | Satellite cell: GAS-R5a | Gastrocnemius muscle (right) | Yes |
| ML7_S43 | Satellite cell: GAS-R5b | Gastrocnemius muscle (right) | Yes |
| ML7_S44 | Satellite cell: GAS-R5c | Gastrocnemius muscle (right) | Yes |
| ML7_S45 | Satellite cell: GAS-R5d | Gastrocnemius muscle (right) | Yes |
| ML7_S46 | Satellite cell: GAS-R5e | Gastrocnemius muscle (right) | Yes |
| ML7_S47 | Satellite cell: EDL-L1 | EDL muscle (left) | Yes |
| ML7_S48 | Satellite cell: EDL-L2a | EDL muscle (left) | Yes |
| ML7_S49 | Satellite cell: EDL-L2b | EDL muscle (left) | Yes |
| ML7_S50 | Satellite cell: EDL-R1a | EDL muscle (right) | Yes |
| ML7_S51 | Satellite cell: EDL-R1b | EDL muscle (right) | Yes |
| ML7_S52 | Satellite cell: EDL-R1c | EDL muscle (right) | Yes |
| ML7_S53 | Satellite cell: EDL-R1d | EDL muscle (right) | Yes |
| ML7_S54 | Satellite cell: EDL-R2a | EDL muscle (right) | Yes |
| ML7_S55 | Satellite cell: EDL-R2b | EDL muscle (right) | Yes |
| ML7_S56 | Satellite cell: EDL-R2c | EDL muscle (right) | Yes |
| ML7_S57 | Satellite cell: EDL-R3 | EDL muscle (right) | Yes |
| ML7_S58 | Islet cell #1 | Pancreas | No |
| ML7_S59 | Islet cell #2 | Pancreas | No |
| ML7_S60 | Islet cell #3 | Pancreas | No |
| ML7_S61 | Islet cell #4 | Pancreas | No |
| ML7_S62 | Islet cell #5 | Pancreas | No |
| ML7_S63 | Islet cell #6 | Pancreas | No |
| ML7_S64 | Islet cell #7 | Pancreas | No |
| ML7_S65 | Islet cell #8 | Pancreas | No |
| ML7_S66 | Islet cell #9 | Pancreas | No |
| ML7_S67 | Oocyte: R1 | Right ovary | No |
| ML7_S68 | Oocyte: R2 | Right ovary | No |
| ML7_S69 | Oocyte: R3 | Right ovary | No |
| ML7_S70 | Oocyte: R4 | Right ovary | No |
| ML7_S71 | Oocyte: R5 | Right ovary | No |
| ML7_S72 | Oocyte: R6 | Right ovary | No |
| ML7_S73 | Oocyte: R7 | Right ovary | No |
| ML7_S74 | Oocyte: R8 | Right ovary | No |
| ML7_S75 | Oocyte: R9 | Right ovary | No |
| ML7_S76 | Oocyte: R10 | Right ovary | No |
| ML7_S77 | Oocyte: R11 | Right ovary | No |
| ML7_S78 | Oocyte: R12 | Right ovary | No |
| ML7_S79 | Oocyte: R13 | Right ovary | No |
| ML7_S80 | Oocyte: R14 | Right ovary | No |
| ML7_S81 | Oocyte: L1 | Left ovary | No |
| ML7_S82 | Oocyte: L2 | Left ovary | No |
| ML7_S83 | Oocyte: L3 | Left ovary | No |
| ML7_S84 | Oocyte: L4 | Left ovary | No |
| ML7_S85 | Oocyte: L5 | Left ovary | No |
| ML7_S86 | Oocyte: L6 | Left ovary | No |
| ML7_S87 | Oocyte: L7 | Left ovary | No |
| ML7_S88 | Oocyte: L8 | Left ovary | No |
| ML7_S89 | Oocyte: L9 | Left ovary | No |
| ML7_S90 | Oocyte: L10 | Left ovary | No |
| ML7_S91 | Oocyte: L11 | Left ovary | No |
| ML7_S92 | Oocyte: L12 | Left ovary | No |
| ML7_S93 | Oocyte: L13 | Left ovary | No |
| ML7_S94 | Oocyte: L14 | Left ovary | No |
| ML7_S95 | Oocyte: L15 | Left ovary | No |
| ML7_S96 | Oocyte: L16 | Left ovary | No |
| ML7_S97 | Oocyte: L17 | Left ovary | No |
| ML7_S98 | Oocyte: L18 | Left ovary | No |
| ML7_S99 | Mesenchymal SC FR1 | Femur (right) | No |
| ML7_S100 | Mesenchymal SC FR2 | Femur (right) | No |
| ML7_S101 | Mesenchymal SC FR3 | Femur (right) | No |
| ML7_S102 | Mesenchymal SC FR4 | Femur (right) | No |
| ML7_S103 | Mesenchymal SC FR5 | Femur (right) | No |
| ML7_S104 | Mesenchymal SC FR6 | Femur (right) | No |
| ML7_S105 | Mesenchymal SC FR7 | Femur (right) | No |
| ML7_S106 | Mesenchymal SC FL1 | Femur (left) | No |
| ML7_S107 | Mesenchymal SC FL2 | Femur (left) | No |
| ML7_S108 | Mesenchymal SC FL3 | Femur (left) | No |
| ML7_S109 | Mesenchymal SC TR1 | Tibia (right) | No |
| ML7_S110 | Mesenchymal SC TR2 | Tibia (right) | No |
| ML7_S111 | Mesenchymal SC TR3 | Tibia (right) | No |
| ML7_S112 | Mesenchymal SC TR4 | Tibia (right) | No |
| ML7_S113 | Mesenchymal SC TR5 | Tibia (right) | No |
| ML7_S114 | Mesenchymal SC TR6 | Tibia (right) | No |
| ML7_S115 | Mesenchymal SC TR7 | Tibia (right) | No |
| ML7_S116 | Mesenchymal SC TL1 | Tibia (left) | No |
| ML7_S117 | Mesenchymal SC TL2 | Tibia (left) | No |
| ML7_S118 | Mesenchymal SC TL3 | Tibia (left) | No |
| ML7_S119 | Oocyte: R15 | Right ovary | No |
| ML7_S120 | Oocyte: R16 | Right ovary | No |
| ML7_S121 | Oocyte: R17 | Right ovary | No |
| ML7_S122 | Oocyte: R18 | Right ovary | No |
| ML7_S123 | Oocyte: R19 | Right ovary | No |
| ML7_S124 | Oocyte: R20 | Right ovary | No |
| ML7_S125 | Oocyte: R21 | Right ovary | No |
| ML7_S126 | Oocyte: R22 | Right ovary | No |
| ML7_S127 | Oocyte: R23 | Right ovary | No |
| ML7_S128 | Oocyte: R24 | Right ovary | No |
| ML7_S129 | Splenic B-Cell #1 | Spleen | Yes |
| ML7_S130 | Splenic B-Cell #2 | Spleen | Yes |
| ML7_S131 | Splenic B-Cell #3 | Spleen | Yes |
| ML7_S132 | Splenic B-Cell #4 | Spleen | Yes |
| ML7_S133 | Splenic B-Cell #5 | Spleen | Yes |
| ML7_S134 | Splenic B-Cell #6 | Spleen | Yes |
| ML7_S135 | Splenic B-Cell #7 | Spleen | Yes |
| ML7_S136 | Splenic B-Cell #8 | Spleen | Yes |
| ML7_S137 | Splenic B-Cell #9 | Spleen | Yes |
| ML7_S138 | Splenic B-Cell #10 | Spleen | Yes |

**Abbreviations:** NK = Natural Killer; SC = Stem Cell; HSC = Hematopoietic Stem Cell; EDL = Extensor Digitorum Longus muscle; SOL = Soleus muscle; GAS = Gastrocnemius muscle; MAS = Masseter muscle;

**Nomenclature of satellite cells:**<SAT>-<*muscle*>-<*body-side*><*fiber #*><*satellite cell #*>

For example: SAT-GAS-R4e is the 5^th^ satellite cell from the fourth fiber of the Gastrocnemius muscle (right side)
