# Supplementary material for: Reconstruction of Cell Lineage Trees in Mice
Source: PLoS One. 2008 Apr 9;3(4):e1939. doi: 10.1371/journal.pone.0001939 (PMC2276688; doi:10.1371/journal.pone.0001939)
Supplement: Text S1 — Algorithm for automatic analysis of capillary electrophoresis signals (0.32 MB DOC) [file pone.0001939.s010.doc]

**Text S1: Algorithm for automatic analysis of capillary electrophoresis signals**

**Overview**

For each sampled cell, a predefined set of microsatellite (MS) loci is amplified (currently about 100) using multiplex PCR, where for every MS locus one of the primers is colored with one of four different fluorescences (FAM, NED, VIC or PET). Then, the amplified products are separated using a Capillary Electrophoresis (CE) machine, while in each run several amplification products (currently up to 12) are separated together, yielding a CE histogram (Figure S1). The goal of the algorithm described here is to analyze these histograms, and determine the relative allelic value of each allele (compared to the corresponding allele in the putative zygote).

**Preprocessing**

The output of the CE for each capillary run is a binary file (.fsa) depicting fluorescentic intensity as a function of time. This file is exported by the GeneMapper program (V3.7) to a text file (called a table file), in which the data has been converted to a series of peaks, each describing an aggregate of DNA fragments sharing a certain length. The position of every peak is given (i.e. an approximation of the fragments’ size in nucleotides), as well as its height, area (both are measures of the aggregate’s relative extent of concentration) and fluorescence.

**A Typical MS Signal**

According to the peak-based representation of data, a typical MS signal is a series of adjacent peaks varying in height and area, where one of them represents the original MS allele (called ‘true peak’), and the others are either a PCR artifact called ‘stutter’ (their distance from the true peak is a whole multiplication of the basic repeat unit length). Another cause for additional peaks is ±A, in which the nucleotide ‘A’ is added to some PCR products. This causes peaks (including stutter) to be divided into subpeaks, one nucleotide apart.

We note that the general shape of a locus’s signal depends highly on the amplification method in use. As a consequence, the confidence levels of both manual and algorithmic analyses tend to vary between easily and problematically analyzed signals. This algorithm is optimized for signals obtained from cloning or from the ‘GenomiPhi DNA Amplification Kit’ (by GE Healthcare).

**Input**

A set of table files. Given in every file is the sample’s name, as well as information regarding the set of loci analyzed in this file, including fluorescence, length of the basic repeat unit and the expected length of the alleles. In addition, for each MS locus in the set, the algorithm receives capillary runs from the tail (representing the putative zygote) and negative control (NC) - ddw.

**Output** (Figure S3)

For each cell, a vector of all relative allelic values/genotypes with length ranging from *n* to *2n* (depending on the amount of mono-allelic loci).

The genotype of an allele can be either one of the following:

- An integer in the range of [-5, 5] representing the number of basic repeat units in which the allele had prolonged/shortened
- ‘X’ if the allele has not been amplified or the confident level of its computational analysis is lower than required
- ‘NCNN’ if the negative control is not negative
- ‘NRV’ (No Root Values) if a MS allele has been identified, but the relative allelic value can not be inferred since no value was set by the algorithm as the length of the allele in the putative zygote

**Method** (Figure S1)

We use a machine learning approach, wherein for each query signal the algorithm utilizes a Nearest Neighbor (i.e. most similar) search in a pre-constructed Training Set (TS), and then analyses the query (meaning, identifies its true peak) in accordance with the chosen TS signal.

**Training Set**

The TS contains up to three prototype signals per locus (depending on the variability of the locus’s signals), and for each signal a pointer to its true peak. The preprocessing of a TS signal is the same as described in section four of the algorithm, except no use is made of the positions vectors.

**
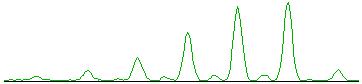

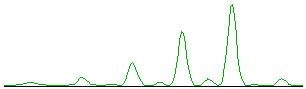

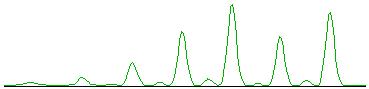

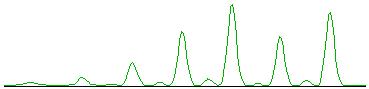

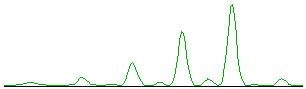

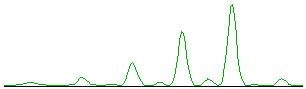
**


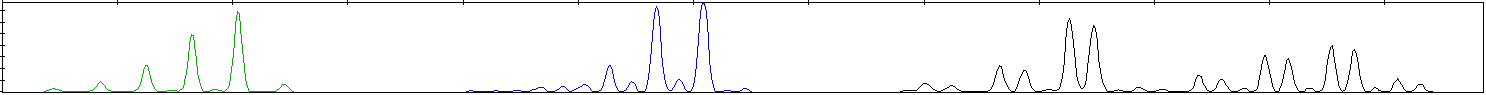

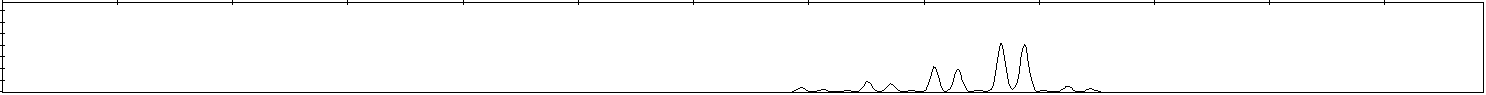

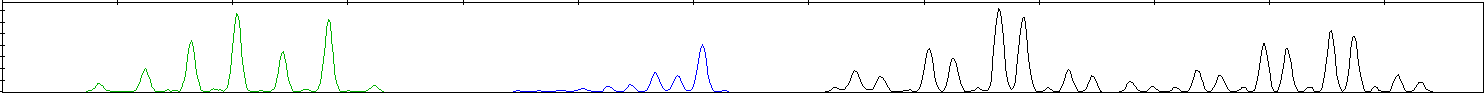


140

145

150

155

160

165

170

195

190

185

180

175


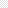

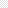


0

0

0

0

0

0

0

+1

-1

0

**Tail**

**Sample**

**NC**

**B**

**A**

Training Set – Locus L1

**Tail**

**TS12**

**TS1 (A)**

**TS2 (A)**

Distance Measurement –

Case of a diallelic signal

Cell Identifier: **[ 0, X, -1, 0, NCNN, NCNN ]**

Locus L1

Locus L2

Locus L3

**Figure S1:** Method overview

**Top**: a capillary histogram of three loci is shown for tail, sample and negative control (NC). Locus L1 in the zygote is a heterozygous di-nucleotide MS, L2 in the zygote is a homozygous di-nucleotide MS and L3 in the zygote is a heterozygous tri-nucleotide MS.

**Lower right**: the two training set (TS) signals of locus L1, for each a pointer shows the true peak.

**Lower left**: a single iteration of the nested loop from the distance measurement that assumes a diallelic signal. Both TS1 and TS2 which the algorithm uses to construct TS12 are actually the same signal (A), and in this iteration the algorithm finds the tail’s nearest neighbor, which will result in the identification of the true peaks as the correct ones.

**Cell identifier**: every pair of genotypes corresponds the locus of the same color. At L1 a single allele is present, which is most likely due to an unamplified allele rather than a mutation of -2 at the longer allele. At L2 one of the alleles has undergone a -1 mutation (as a rule of thumb when analyzing homozygous the genotypes are presented in an ascending order). At L3 both alleles are less than 7 repeat units away from the NC signal and no more than 5 times its height, therefore their genotypes are discarded.

X

**Algorithm**

Note that we use ‘BRUL’ as an abbreviation of Basic Repeat Unit’s Length, e.g. for a mono-nucleotide locus BRUL = 1, for a di-nucleotide locus BRUL = 2, etc. The recommended BRUL for the algorithm is two (di-nucleotides) and above. Mono-nucleotides can also be analyzed, though the confidence level of the analysis would be lower.

Execute the following series of commands on the tail table files, NC table files and sample table files in the given order:

For each locus:

1. Isolate the region from the whole graph (w.r.t the X axis) where the signal is expected to be found.

*The range is defined as follows:*

*[length of shorter allele – 10 BRUL , length of longer allele + 7BRUL]*

*Note that in the tail's analysis, the algorithm uses the alleles' estimated length (given as input), and in NC and samples' analyses it uses the accurate lengths of the alleles from the tail, as obtained from the tail’s analysis. Hence the tail is the first to be analyzed. Also, the algorithm assumes that signals from the same capillary that share the same fluorophore are sufficiently distant from one another.*

1. Discard peaks which most likely have not originated from a MS.

*An unlikely peak is one that does not conform to the following linear relation:*

*peak’s height peak’s area 0.185 + 450*

1. Cluster adjacent peaks to form separated signals (each signal will be considered as a potential MS allele).

*The criterion for clustering two adjacent peaks is:*

*distance between peaks 1.3BRUL*

1. For each signal/potential MS allele:
   1. Represent the signal in the form of two vectors (Figure S2):
      1. *Construct a vector that holds the positions of the peaks, by setting the positions as the values of the vector so that the distance between their locations on the vector (i.e. the difference between their indices) will be equal to the rounded distance between the peaks’ positions.*
      2. *Construct a vector that holds the heights of the peaks according to the positions vector, by setting the height of each peak under the corresponding index of its position on the positions vector, and set all other values to be zero.*
      3. *Remove from both vectors all of the peaks which are not true/stutter, thus creating two shorter vectors, in the following manner:*

*Identify the highest peak in the heights vector and all other peaks whose indices on the vector differ from the index of the highest one by a whole multiplication of BRUL, then discard all of the other peaks from both vectors.*

- - 1. *Divide all of the values in the heights vector by the maximal height, to form a vector with values ranging from zero to one.*
  1. Perform a nearest neighbor search, assuming that the signal has originated from a single allele, and deduce its shift (see Distance Measurement – Case of a monoallelic signal).
  2. Perform a nearest neighbor search, assuming that the signal has originated from a pair of close alleles, and deduce both of its shifts (see Distance Measurement – Case of a diallelic signal).

**Clarification**: As for now, each signal in the region of interest holds the potential to eventually be identified by the algorithm as a single MS allele or as a pair of close alleles.

- 1. Disqualify the signal from being a potential single allele or a potential pair of alleles (separately) in case that it fails to fulfill any of the following criteria:

Note that for signals obtained from different origins (tail/NC/samples) different criteria are applied (see the parenthesis in the beginning of each criterion). Also, unless mentioned otherwise the criterion is applied to both the potential of being a single allele and a pair of alleles.

- - 1. *(Tail, NC, Samples) The match score/distance must be 0.5 at most.*
    2. *(Tail, Samples) The original height of the highest peak in the signal must be at least 50.*
    3. *(Tail, NC, Samples) The shift of the allele must be $\pm5.1\times\text{BRUL}$ at most.*
    4. *(Tail, Samples) For the signal to be a potential pair of alleles, the locus must be diallelic in the first place.*
    5. *(Tail, NC, Samples) If BRUL3, a signal of a single allele must have at least 3 peaks and a signal of a pair of alleles must have at least 4 peaks.*

*If BRUL >3, a signal of a pair of alleles must have at least 3 peaks (no constraint on a single allele).*

- - 1. *(Tail, Sample) For a signal of a pair of alleles, if the analysis shows that the two ‘true’ peaks differ in a single repeat unit, the shorter/left one must be higher than the longer/right one.*

1. Choose the most likely genotype of the locus’s allele/alleles assuming the following model of MS behavior:

- *If |*a*| < |*b*| then a shift of* a *repeat units is more probable than a shift of* b *repeat units.*
- *If* a *> 0 then a shift of* –a *repeat units is more probable than a shift of* a *repeat units (i.e. shortening is more probable than elongation).*
- *If in the zygote allele A1 is longer than allele A2, it can not become shorter than A2 in any of the organism’s cells (no cross between alleles).*
- *In case of more than two potential alleles, the pair with the minimal sum of mutations (in absolute value) is the most probable.*

1. (For tail files only) Allow manual validation of the analysis.

The goal of the manual validation is to avoid continuous erroneous analyses in all of the corresponding alleles from the cellular samples, and to overcome cases of unamplified alleles in the tail.

1. (For Sample files only) Change the genotype to ‘NCNN’ if the NC is not negative, according to the following criterion:

*If a MS allele has been identified in the NC, and:*

*distance between sample allele and NC allele 6BRUL, and:*

*height of sample allele 5*  *height of NC allele.*

1. (For Sample files only) Change the genotype of alleles which deviate from their expected positions to ‘X’, according to the following criterion:

*If the sample’s true peak is more than 0.5bp away from its expected position (e.g. if in a di-nucleotide mono-allelic locus, the true peak of the allele in the tail is at X=200, the true peak of the sample’s allele is not likely to be at 203).*

1. (For Sample files only) If the amplification method used is one of the recommended methods (cloning/GenomiPhi) and BRUL2 also as recommended, issue a warning for shifts higher than or equal to 3 and to shifts lower than or equal to -2. Otherwise, suggest a manual override on all of the analysis as a whole.

Color Position Height Area

G 139.02 484 2864

G 141.01 1230 7630

G 142.04 101 453

G 143.07 2322 19644

G 144.10 109 484

G 145.05 3445 52408

G 146.00 85 320

G 146.98 1521 17713

G 148.03 120 804

G 149.00 3310 47330

G 150.02 78 398

G 151.10 380 2847

**484**

78

**3310**

**420**

120

**1521**

85

**3445**

109

**2322**

101

**1230**

0

139.02

150.02

149.00

151.10

148.03

146.98

146.00

145.05

144.10

143.07

142.04

141.01

-


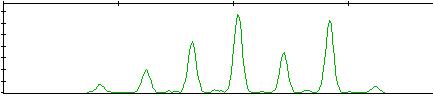


135

140

145

150

3000

2000

1000

0

**Heights**

**Stutter’s Heights**

**Positions**

Insertion

**Input file**

**CE Signal**

**0.14**

**0.96**

**0.12**

**0.44**

**1.00**

**0.67**

**0.36**

**484**

**3310**

**420**

**1521**

**3445**

**2322**

**1230**

**Figure** **S2**: Representing a signal as two vectors.

Initially, the output of the CE undergoes a preprocessing stage and is converted from a binary file depicting fluorescentic intensity as a function of time (not shown) into a text file wherein the data is represented as a series of peaks, each describing an aggregate of DNA fragments (see ***Input file***, ***MS signal***). Then, the data in the positions column of every potential MS allele is transformed into vector (see ***Positions***), wherein each value represents the position of a single peak, and the distance between their locations on the vector (i.e. the difference between their indices) is equal to the rounded distance between the peaks’ positions. E.g. the positions of the first two peaks in the input file differ by: round(141.01-139.02) = round(1.99) = 2, and therefore will be placed 2 indices apart from each other on the vector.

The heights vector is constructed out of the heights column according to the positions vector (each peak’s height in the same index as its position), and all other values are set to zero (see ***Heights***). Afterwards, all peaks which are not true/stutter are discarded from the heights vector by finding the highest peak and discarding all peaks which their distance from the highest is not a whole multiplication of BRUL (see ***Stutter’s heights***). Finally, all values in the heights vector are divided by the height of the highest peak (see ***Normalized Stutter’s Heights***).

**Normalized Stutter’s Heights**

**Distance Measurement**

We use $Q_{i}$and $\text{TS}_{i}$to denote the values in the query and TS heights vectors (respectively) at index *i*.

We define the operation of ‘normalizing’ *TS* according to *Q* w.r.t the specific shift between the vectors in the time of normalization, in the following manner:

1. Find the highest value/peak in the heights vector *TS* (assume that its index is *k*).
2. Find the height of the corresponding peak in the heights vector *Q* (the one with index *k*). We denote this height with *N* (*N =*$Q_{k}$).
3. Multiply all of the values in *TS* by *N*.

**Case of a monoallelic signal**

For each single training set signal that belongs to this locus (denoted by *TS*):

For each possible shift between *TS* and *Q*:

- Normalize *TS* according to *Q* (norm. coefficient is *N*).
- Compute the distance between *Q* and *TS* according to the following metric:

Choose the minimal distance obtained from this search as the final distance, and deduce the position (X value) of the query’s true peak according to the chosen training set signal’s true peak.

**Case of a diallelic signal** (Figure S1)

For each pair of training set signals that belongs to this locus (denoted by$\text{TS}1$and$\text{TS}2$):

For each possible shift between$\text{TS}1$and *Q*:

- Normalize$\text{TS}1$ according to *Q* (norm. coefficient is *N1*).

For each possible shift between$\text{TS}2$and *Q*:

- Normalize$\text{TS}2$ according to *Q* (norm. coefficient is *N2*).
- Compute the distance between *Q* and $\text{TS}12$using the following metric: , where:

Choose the minimal distance obtained from this search as the final distance, and deduce the positions (X values) of the query’s true peaks according to the chosen training set signals’ true peaks.

**Override of algorithm output**

1. Manual override is suggested for signals that are defined by the algorithm as problematic. As discussed above, currently all signals which underwent WGA amplification using the Genome-Plex kit are defined as problematic
2. Alleles in which all analyzed signals (at least two) are mutations are currently overridden to Problematic Signal (PS). Although it is possible that all samples are mutated at a certain allele, it is also possible that the reference was incorrectly analyzed, meaning that all mutations are actually incorrect. We became aware of this possibility when two independent WGAs of tail DNA yielded different allelic values in a few cases (meaning that WGA may introduce mutations even in bulk DNA). We are currently investigating this issue, yet because in general we prefer to assign mutations only when we are certain about them, we currently override mutations in this situation.

**Algorithm Benchmarking**

In order to evaluate the algorithm we have performed a comparison between the computational analysis and the manual analysis of two sets of cells. The first is taken from a 5.5 weeks old mouse (ML7), where the percentage of recorded mutations is relatively low, and the other is taken from a 40 weeks old mouse (ML8; Frumkin D. *et al.*, manuscript in preparation), where the percentage of mutations is relatively high. In both experiments the amplification method is GenomiPhi. The results are:

Benchmarking summary of mouse ML7

Num of compared signals = 15,825

Num of warnings = 284

Table 1: Comparison between manual and automatic analysis of ML7

(We use **Mutation M1** and **M2** to denote different mutations, e.g. if M1=1, then M2 is neither 0 nor 1)

|  | | **Automatic** | | | |
| --- | --- | --- | --- | --- | --- |
|  |  | **0 (no mutation)** | **Mutation M1** | **Mutation M2** | **X (dropout)** |
| **Manual** | **0 (no mutation)** | **6,135** | **71** | **0** | **1,919** |
|  | **Mutation M1** | **46** | **534** | **9** | **429** |
|  | **X (dropout)** | **291** | **77** | **0** | **6,314** |

Benchmarking summary of mouse ML8

Num of compared signals = 6,438

Num of warnings = 283

Table 2: Comparison between manual and automatic analysis of ML8

**(We use Mutation M1 and M2 to denote different mutations, e.g. if M1=1, then M2 is neither 0 nor 1)**

|  | | **Automatic** | | | |
| --- | --- | --- | --- | --- | --- |
|  |  | **0 (no mutation)** | **Mutation M1** | **Mutation M2** | **X (dropout)** |
| **Manual** | **0 (no mutation)** | **990** | **21** | **0** | **286** |
|  | **Mutation M1** | **10** | **559** | **2** | **197** |
|  | **X (dropout)** | **57** | **44** | **0** | **4,272** |

**Output Examples**

1. An automatic signal analysis.


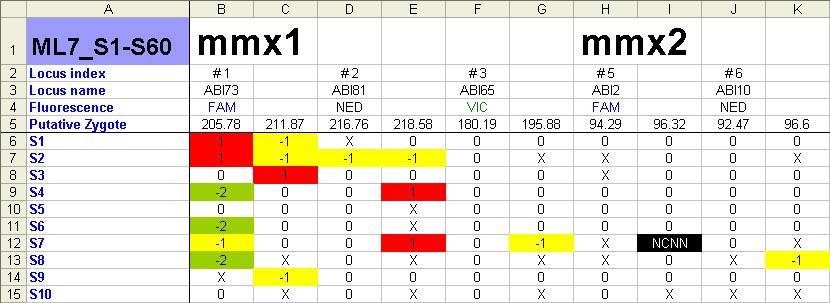
**Figure S3:** An output example of signal analysis performed by the algorithm.

Each row represents a single cell and each column represents a single allele. Every mutation is colored uniquely, and ‘NCNN’ is colored in black. The ‘mmx #’ marks the number of the PCR multiplex group in which the loci beneath it have been amplified.

1.
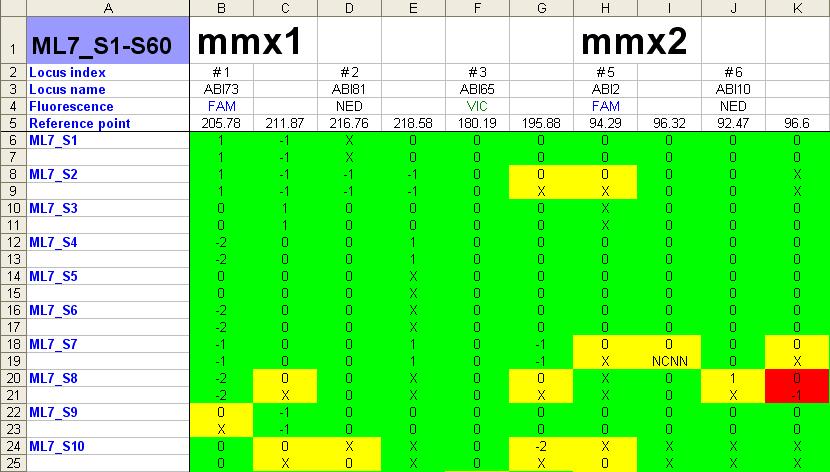
A comparison between manual and automatic analyses.

**Figure S4**: An output example of a comparison between the previously presented (Figure #2) automatic analysis and the corresponding manual analysis.

In each pair of rows the upper is the manual analysis and the lower is the automatic analysis.

Legend: Green - a successful analysis. Red - an error that is most likely to have a significant effect on the downstream procedure (e.g. manual = ‘0’, automatic = ‘2’). Yellow – an error that would most likely not have a significant effect on the downstream procedure (e.g. manual = ‘X’, automatic = ‘0’) or a ‘loss of data’ error (e.g. manual = ‘1’, automatic = ‘X’).
